# A McAb-Based Direct Competitive ELISA to Detect O:9 *Salmonella* Infection in Chicken

**DOI:** 10.3389/fvets.2020.00324

**Published:** 2020-07-03

**Authors:** Zemiao Xia, Haopeng Geng, Yuan Cai, Yaonan Wang, Daquan Sun, Jian Zhang, Zhiming Pan, Xin'an Jiao, Shizhong Geng

**Affiliations:** ^1^Jiangsu Co-innovation Center for Prevention and Control of Important Animal Infectious Diseases and Zoonoses, Yangzhou University, Yangzhou, China; ^2^Jiangsu Key Laboratory of Zoonosis, Yangzhou University, Yangzhou, China; ^3^Key Laboratory of Prevention and Control of Biological Hazard Factors (Animal Origin) for Agrifood Safety and Quality, Ministry of Agriculture of China, Yangzhou University, Yangzhou, China; ^4^Joint International Research Laboratory of Agriculture and Agri-Product Safety of the Ministry of Education, Yangzhou University, Yangzhou, China

**Keywords:** O:9 *Salmonella*, McAb, O_9_ Dc-ELISA, specificity, PAT

## Abstract

*Salmonella enteritidis* and *Salmonella pullorum* belonging to Group O_9_ *Salmonella* are major causative agents of infectious diseases in chicken. O_9_ antigen as a part of lipopolysaccharide (LPS) is a predominant detected target for *Salmonella* infection. To identify the infection, an anti-O_9_ monoclonal antibody (McAb)-based direct competitive enzyme-linked assay (O_9_ Dc-ELISA) was developed after constraints were optimized; the establishment and application of O_9_ Dc-ELISA, compared to two commercial kits and plate agglutination test (PAT), showed that O_9_ Dc-ELISA could screen out more positive samples than the PAT method could and produce the same agreement rates with commercial kits in terms of sensitivity in addition to strong specificity to clinical serum samples.

## Introduction

*Salmonella*, an important zoonotic pathogen, is one of the major causative agents of food-borne infectious diseases worldwide (1). Consumption of foods such as egg, chicken, pork, beef, and dairy products contaminated with *Salmonella* can cause salmonellosis in humans (2–4). This pathogen not only brings huge economic loss in the animal industry but also impacts human health, even death (5–8). Because of these disease harms and public health hazards, efficient surveillance is very important to reduce the prevalence of *Salmonella* and the risk of transmission to humans.

*Salmonella enteritidis* and *Salmonella pullorum*, which are important members in group O:9 *Salmonella*, are the main pathogens found in modern large-scale chicken farms in China (2, 9–11). In addition to their high morbidity and mortality in young broilers, they cause non-apparent infections in adult chickens without obvious clinical symptoms. Thus, it is difficult to find *Salmonella* infection in adult chickens. If *Salmonella*-infected chicken is not found on time, it may be a source of infection causing unlimited spread in chicken, even to humans, because of horizontal transmission and vertical transmission. It is necessary to carry out a seroepidemiological survey on *Salmonella* for healthy breeding and food safety.

Currently, plate agglutination test (PAT) is the main detection method used during *Salmonella* surveillance for its easy operation and low cost, but its sensitivity and specificity are poor and can easily cause false results because of antigen detection, visual observation, and subjective judgment.

LPS is the main antigen found on the *Salmonella* surface and the primary target for the immune system (12). After *Salmonella* infection, LPS can induce and keep a high level of antibody from early stages. Serotyping using serum/antibodies to the O-antigen of *Salmonella* lipopolysaccharide (LPS) (13, 14) is a critical basis of current *Salmonella* surveillance programs. Routine serotyping helps in monitoring public health response to the global challenge of salmonellosis and the effectiveness of control measures (9, 15–17).

Therefore, the development of readily available detection systems of the *Salmonella* antibody in chicken is important for mass-scale laboratory diagnosis. In this study, we developed an anti-O:9 *Salmonella* McAb-based direct competitive ELISA method to meet the requirements of accurate *Salmonella* surveillance.

## Materials and Methods

### Ethical Statement

The present study was conducted under the approval of Laboratory Animal Ethics Committee of Yangzhou University (Jiangsu province, China) in accordance with Laboratory Animal Guidelines for ethical review of animal welfare (GB/T 35892-2018, National Standards of the People's Republic of China).

### Strain, Hybridoma Cell Line, and Animals

*Salmonella enteritidis* (C50041) and *Salmonella pullorum* (S06004) were stored by our laboratory. A 3-47-0 hybridoma cell line secreting anti-O_9_ McAb was developed and preserved by our laboratory. Thirty 10-week BALB/c female mice were purchased for ascites from Comparative Medical Center of Yangzhou University.

### Primary Quantity of Coated LPS and HRP-Labeled O_9_ McAb for Competitive ELISA

Primary quantities of LPS and HRP-labeled O_9_ McAb were confirmed by chessboard titration to develop a direct ELISA following conventional ELISA protocol. Horizontal gradient dilution of HRP-labeled O:9 McAb and vertical gradient dilution of coating antigens were performed. The final concentration of a tested positive serum was diluted 1:10. According to the serum inhibition rate [inhibition rate = (1-detected serum OD value/blank control OD) ×100%], optimal balanced concentrations were selected.

### Constraint Optimizations for O9 Dc-ELISA

#### Tested Positive Serum Dilution

Two *Salmonella pullorum*-positive sera, two *S. enteritidis*-positive sera, and two negative sera from specific pathogen-free (SPF) chicken were diluted 1:4, 1:8, 1:16, 1:32, 1:64, 1:128, and 1:256. Based on the previous direct ELISA, each tested serum dilution was used as a competitor of positive serum and subjected to a competition ELISA. The serum dilution at the highest inhibition rate was selected as the serum dilution of the competitive ELISA method.

### Quantity of Coated LPS and HRP-Labeled O:9 McAb

Based on the previous ELISA, LPS were divided into four groups, 480, 320, 190, and 160 ng/mL. By comparing the N/P values (negative serum OD value/positive serum OD value), the coating concentration at which the N/P value was the largest was chosen out as the optimal concentration.

Similarly, the antibody was divided into six groups, 56.8, 52.0, 48.0, 44.6, 41.6, and 39.1 ng/mL, to optimize the concentration of HRP-labeled McAb. The N/P values were compared (negative serum OD value/positive serum OD value) with the concentration of the HRP-labeled O_9_ McAb.

### Time of LPS Being Coated Onto a Plate

Three ELISA plates were coated at 100 μL/well at an optimized coating concentration. The coating time of the three ELISA plates was 16, 24, and 36 h, respectively.

### Time of HRP-labeled McAb Binding With LPS and Reacting With TMB

Competitive ELISA was performed with the LPS coating concentration and HRP-labeled McAb and serum dilution, which were optimized in the previous steps. To ensure McAb to bind with coated LPS as possible, the incubation time of the HRP-labeled McAb was set to 1.0, 1.5, 2.0, 2.5, and 3.0 h, respectively, for analysis based on the N/P value.

After optimization time of HRP-labeled McAb binding with LPS, the incubation time of HRP-labeled McAb to react with substrate 3,3′,5,5′-tetramethylbenzidine (TMB) was also optimized. The hydrolysis time for TMB substrate by HRP was set to 3, 5, and 10 min.

### Setting Up of the Cutoff Value and Comparison With Commercial Kits and PAT

One hundred serum samples from artificially infected chickens at different time points as positive control and 100 serum samples from SPF chickens as negative control were detected using the France ID.vet *Salmonella* kit, and these 200 serum samples were detected by O_9_ Dc-ELISA; the receiver operating characteristic (ROC) curve was made according to the inhibition rate. Based on these results, the cutoff value which was the value of negative samples + 3SD as a negative/positive judgment boundary was set up.

Fifty random clinical serum samples were tested using a double blind test by O_9_ Dc-ELISA and compared to IDEXX ELISA kit (IDEXX USA, 99-0002040) and ID.vet ELISA kit (ID.vet France, SALSGPD-5P) to judge the accuracy of O_9_ Dc-ELISA in clinical application.

The coincidence rate was calculated by the following formula: number of [(+,+) + (-,-)]/total number %.

### Statistical Analysis

Statistical analysis was performed using GraphPad Prism 5 (GraphPad Software, USA). One-way ANOVA followed by Dunnett's multiple-comparison tests was used to determine the statistical differences between multiple experimental groups. All data are expressed as mean ± standard error of the mean (SEM) unless otherwise specified. *P* < 0.05 was considered statistically significant. ^*^p < 0.05, ^**^p < 0.01, ^***^p < 0.001, ^****^p < 0.0001.

## Results

### Preparation of Coated LPS and HRP-Labeled O_9_ McAb

**Figure 1:**
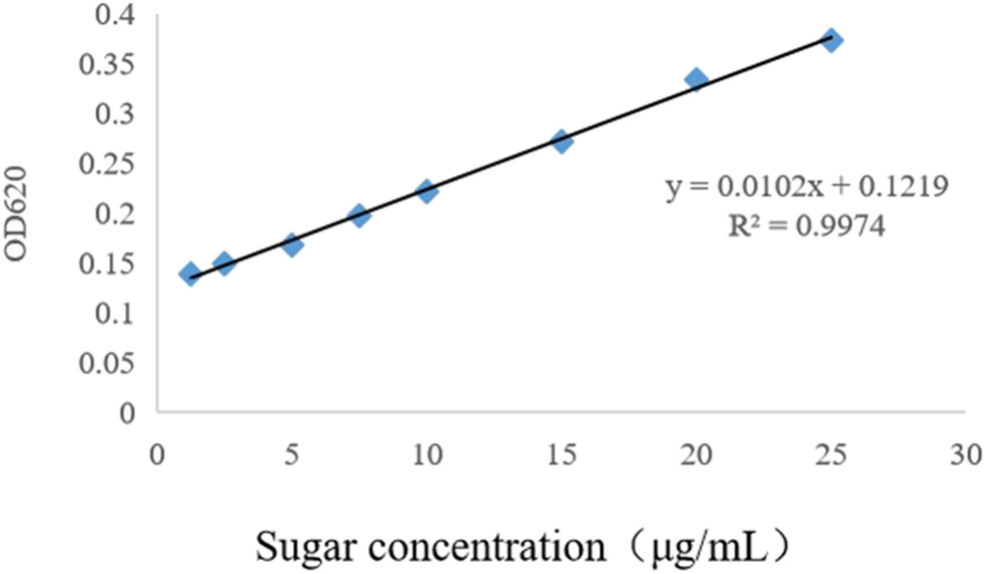
Standard curve of sugar concentration determined by the anthracene-ketone method.

In this study, LPS were purified by the hot phenol–water method (18) and its concentration was calculated according to the standard sugar curve made by the anthracene ketone method. According to the measured OD_620nm_ value and standard curve (Figure 1), the final concentration of LPS was 484.31 μg/mL. McAb against O_9_ LPS (O_9_ McAb) was purified from hybridoma supernatants by caprylic/ammonium sulfate precipitation (19) and labeled with horseradish peroxidase (HRP) (20). The titer of HRP-labeled O_9_ McAb (HRP-O_9_ McAb) was up to 51,200 by indirect ELISA.

### Constraint Determination of Competitive ELISA

**Figure 2:**
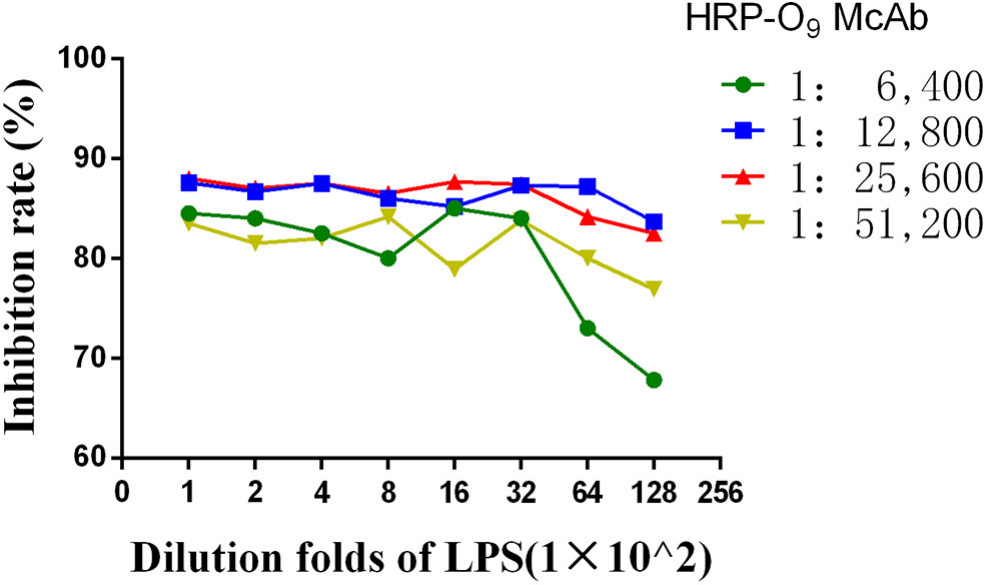
Curves of the HRP-labeled McAb binging to coated LPS.

**Figure 3:**
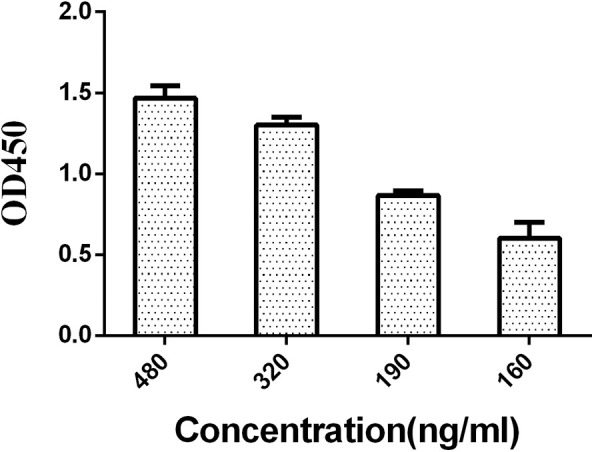
Concentration optimization of coated LPS in O_9_ Dc-ELISA.

**Figure 4:**
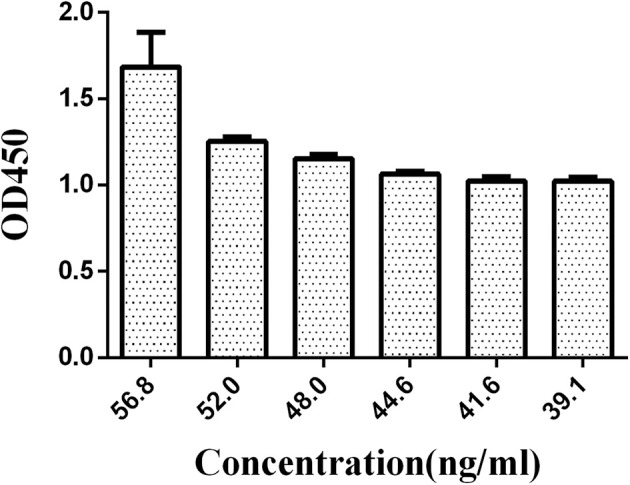
Concentration optimization of HRP-labeled O_9_ McAb in O_9_ Dc-ELISA.

**Figure 5:**
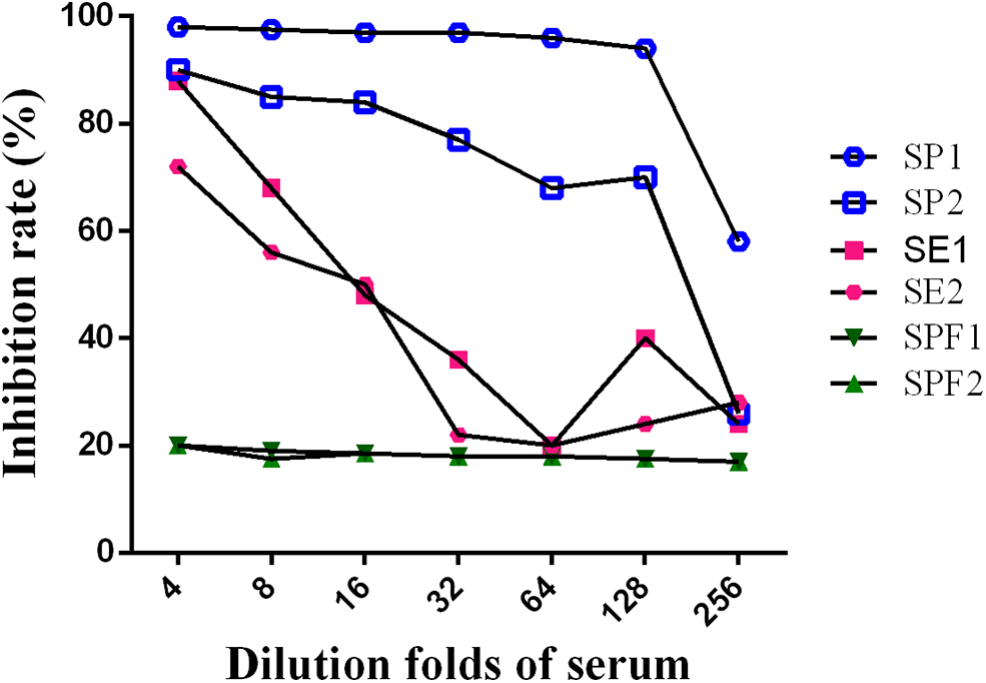
Optimization of positive serum dilution. (SE, SE2: serum by *Salmonella enteritidis* 1, 2; SP1, SP2: serum by *Salmonella pullorum* 1, 2; SPF1, SPF2: serum from SPF chickens).

**Figure 6:**
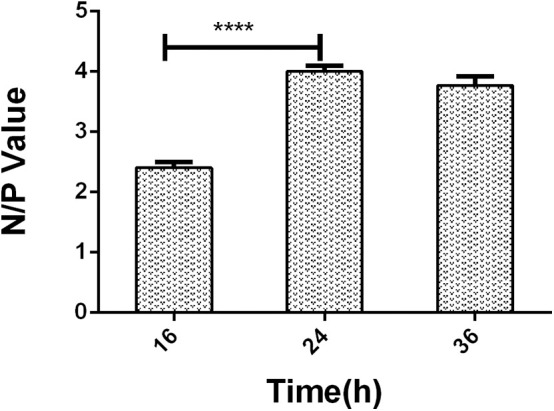
The negative/positive OD value of different LPS coating time in O_9_ Dc-ELISA. ****p < 0.0001.

**Figure 7:**
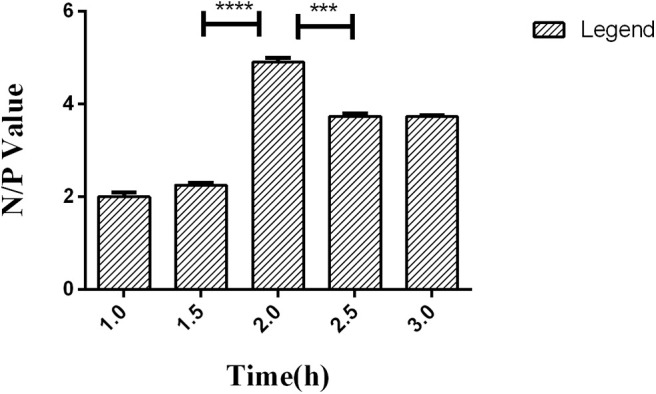
Time effect of HRP-labeled O_9_ McAb binding with coated LPS in O_9_ Dc-ELISA based on the negative/positive OD value. ***p < 0.001, ****p < 0.0001.

**Figure 8:**
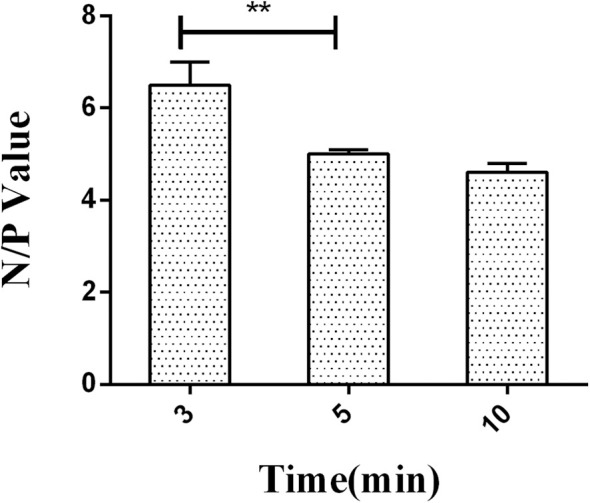
The negative/positive OD value of different reactive times of HRP labeled on O_9_ McAb with substrate TMB. **p < 0.01.

A series of dilutions of LPS, HRP-labeled O_9_ McAb, and positive sera were prepared for chessboard titration and optimization (Figure 2). After optimization assay, 320 ng/ml LPS for coating (Figure 3), 41.6 ng/ml HRP-labeled O_9_ McAb (Figure 4), and positive serum dilution of 1:4 (Figure 5) were selected for developing O_9_ Dc-ELISA. The inhibition rate of positive serum by *Salmonella pullorum* and *Salmonella enteritidis* was up to 94 and 89%, respectively. On this basis, a standard operating procedure was formulated, after 96-well plates (Biofil company, Canada, FEP101896) were coated with ~100 μL purified LPS (320 ng/ml) in carbonate bicarbonate buffer (CBS, pH 9.4) at 4°C for 24 h (Figure 6) and washed with PBST (0.05% Tween 20 in phosphate-buffered saline) two times; 200 μL/well 2% BSA PBS solution was added again for blocking for 3 h at 37°C, then 50 μL 1:2 diluted chicken serum (PBS for blank control) and 50 μL HRP-labeled O_9_ McAb of 41.6 ng/ml were added at the same time. After incubation at 37°C for 2 h (Figure 7), all unbound materials were removed by washing with PBST six times. 100 μL of TMB chromogenic substrate was added to each well and incubated at 37°C for 3 min (Figure 8). After the color development was completed, 50 μL of 2 M H_2_SO_4_ was added to each well to terminate the color development, and the OD450_nm_ absorption value was read.

### Specificity Analysis of O_9_ Dc-ELISA

**Figure 9:**
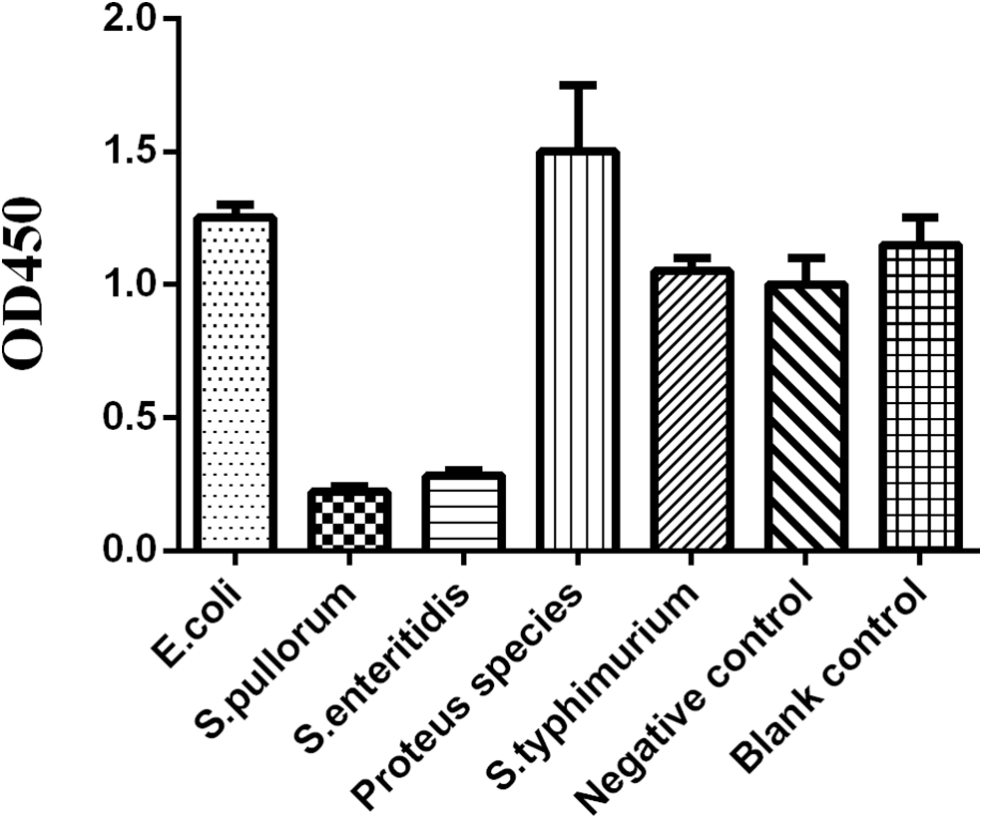
The OD value of chicken sera against different pathogens for specificity analysis.

We prepared the tested sera from chicken infected by *Escherichia coli, Proteus mirabilis*, non-O_9_ *Salmonella* [*Salmonella typhimurium* (O:4)], the negative sera from SPF chickens, and the positive sera from chickens infected with *Salmonella pullorum* and *Salmonella enteritidis*; the results showed that O_9_ Dc-ELISA could not check out the sera against non-*Salmonella* and non-O_9_ *Salmonella*. The value of negative sera was more than 1.0 whereas the OD value of positive sera was less than 0.25 as a control (Figure 9) based on P/N≥2.1.

### Setting Up of the Cutoff Value

**Figure 10:**
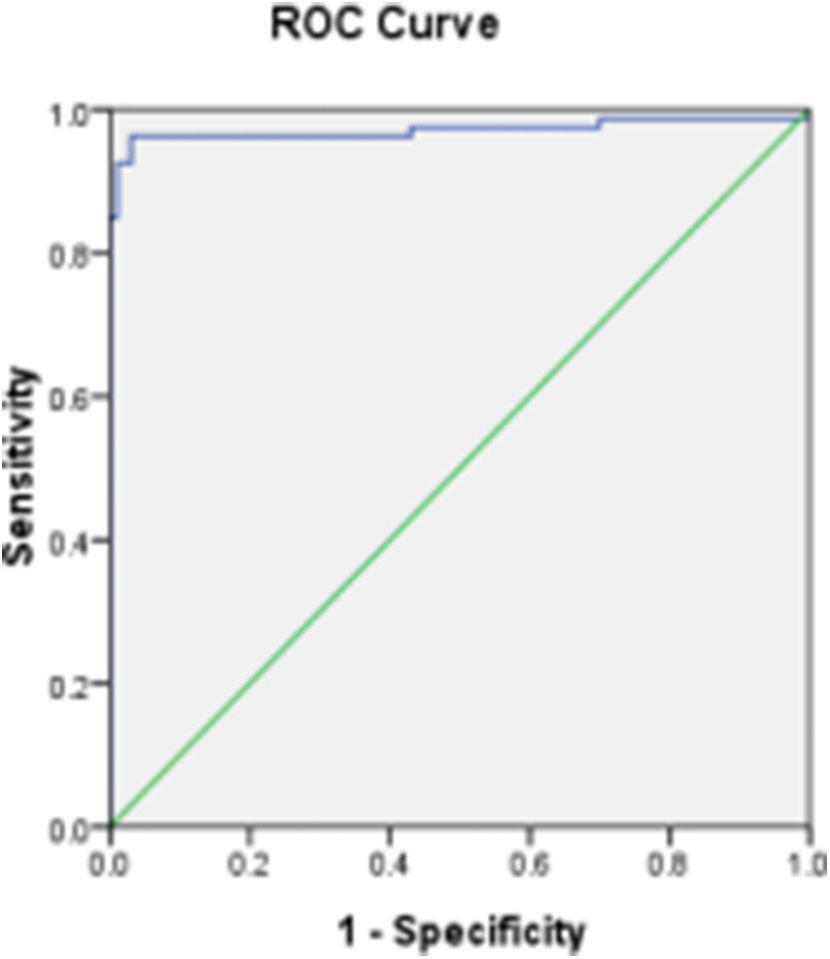
The distribution of inhibiting rate by Dc-ELISA and setting up of the cut-off value.

**Figure 11:**
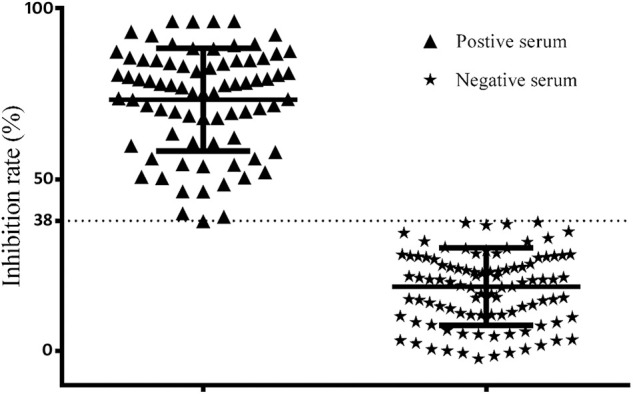
ROC analysis curve for competitive ELISA detecting chicken serum samples.

According to the results using the France ID.vet *Salmonella* kit as a reference of positive and negative chicken sera, and the percent inhibition (PI) values by O_9_ Dc-ELISA which were calculated using the formula PI (%) = (1−OD_450_ of test serum/OD_450_ of blank control) ×100%, the cutoff based on the ROC curve was 38% (Figure 10). Under PI of 38%, the specificity of O_9_ Dc-ELISA reached up to 99.7% and the sensitivity reached up to 96.2% in ROC. The distribution of 100 positive serum samples and the 100 negative serum samples determined by O9 Dc-ELISA showed that 38% of inhibiting rate was indeed a threshold which could distinguish positive serum and negative serum (Figure 11).

### Comparison Among O_9_ Dc-ELISA, Three Commercial Kits, and PAT

To validate the test ability of O_9_ Dc-ELISA, we randomly collected 50 serum samples for comparison to the results using O_9_ Dc-ELISA, PAT, IDEXX ELISA kit, and ID.vet ELISA kit; the results revealed that their coincidence rates were 88% (44/50, Table 1), 98% (49/50, Table 2), and 98% (49/50, Table 3), respectively. Although four samples negative with PAT were positive with O_9_ Dc-ELISA and two commercial ELISA kits, and 1 sample negative with O_9_ Dc-ELISA, IDEXX, and ID.vet ELISA kit was positive with PAT, there was no statistical difference among 4 methods. The results showed that O_9_ Dc-ELISA could screen out more positive samples than the PAT method could and produced the same agreement rates with two commercial kits in terms of sensitivity in addition to strong specificity.

**Table 1:** Comparison of the results between O_9_ Dc-ELISA and PAT.

		**PAT**		**Total**
		**+**	**-**	
O_9_ Dc-ELISA	+	1	4	5
	-	2	43	45
	Total	3	47	50

**Table 2:** Comparison of the results between O_9_ Dc-ELISA and IDEXX ELISA kit.

		**IDEXX ELISA Kit**		**Total**
		**+**	**-**	
O_9_ Dc-ELISA	+	4	1	5
	-	0	45	45
	Total	4	46	50

**Table 3:** Comparison of the results between O_9_ Dc-ELISA and ID.vet ELISA kit.

		**ID.vet ELISA Kit**		**Total**
		**+**	**-**	
O_9_ Dc-ELISA	+	4	1	5
	-	0	45	45
	Total	4	46	50

## Discussion

*Salmonella enteritidis* and *Salmonella pullorum* are two of the most important *Salmonella* spp. that threaten the poultry industry, and humans are infected by directly or indirectly eating contaminated water and food, which causes great hazard to human public health security (21, 22). In our study, a McAb-based competitive ELISA was established to detect O:9 *Salmonella* infection in chicken. In order to achieve a better reaction system, we explored various conditions, including concentration of LPS coating and HRP-labeled O_9_ McAb, serum dilution, LPS coating time, and reaction time of HRP-labeled McAb.

In order to confirm that the established O_9_ Dc-ELISA did not cause a cross-reaction, we used O_9_ Dc-ELISA to test *Escherichia coli, Proteus mirabilis, Salmonella typhimurium* (O:4), and negative sera from SPF chicken, *Salmonella pullorum*, and *Salmonella enteritidis*. The tests showed that only sera from *Salmonella pullorum* and *Salmonella enteritidis* could cause significant inhibition.

By testing 100 artificial positive samples and 100 negative serum samples from SPF chickens and 50 random clinical serum samples, the sensitivity and specificity at different thresholds were compared, and the final selected inhibition rate was 38% as the critical value of the competition ELISA kit. According to ROC, the specificity of O_9_ Dc-ELISA was 99.7%, and the sensitivity was 96.2%. This O_9_ Dc-ELISA was compared with PAT, IDEXX ELISA kit, and ID.vet ELISA kit, respectively. The results showed that the coincidence rate of the O_9_ Dc-ELISA kit and ID.vet ELISA kit was 98%; the coincidence rate with the *Salmonella enteritidis* test kit was 98%; and the coincidence rate with PAT was 88%. The above results indicated that this O_9_ Dc-ELISA has a good detection effect on the O_9_ antibody and had better performance than the PAT method based on more positive samples being checked out and the same agreement rates with commercial kits in terms of sensitivity in addition to strong specificity in the detection of clinical samples. This kit offered a good base as a first-generation product; it will be further evaluated and optimized according to clinical detection performance based on more serum samples to develop a second-generation kit in the future.

## Conclusion

O_9_ Dc-ELISA has good ability in O_9_ antibody detection and had better performance than the PAT method and agreement rates with commercial kits in terms of sensitivity during the detection of clinical chicken serum samples. It must play an important role in O:9 *Salmonella* detection for *Salmonella* clearance in China in the future.

## Data Availability Statement

All datasets generated for this study are included in the article/supplementary material.

## Ethics Statement

The animal study was reviewed and approved by the Animal Welfare and Ethics Committees of Yangzhou University.

## Author Contributions

SG, XJ, and HG designed the paper. HG, ZX, and DS performed the experiments. YC, JZ, and YW provided help during experiments. ZP and XJ made critical revisions to the paper and contributed to paper writing. All authors contributed to the article and approved the submitted version.

## Conflict of Interest

The authors declare that the research was conducted in the absence of any commercial or financial relationships that could be construed as a potential conflict of interest.
